# Supplementary material for: A user-centred design framework for mHealth
Source: PLoS One. 2020 Aug 19;15(8):e0237910. doi: 10.1371/journal.pone.0237910 (PMC7444488; doi:10.1371/journal.pone.0237910)
Supplement: Attachment — Submitted filename: Response to Reviewers.docx [file pone.0237910.s001.docx]

We thank the editor and the reviewers for their valuable comments. We explain below how we have addressed them to improve the clarity of the manuscript.

| Editor’s Comments | Response to comments |
| --- | --- |
| 1. the goal of the paper is unclear – is this to present a new framework for the design of mHealth applications and if so then the rigor around the development of this framework is needed. | The goal of the paper is to address the lack of mHealth deign frameworks for low-resource contexts. The following text has been added:  “The goal of the paper is to explore a combination of user-centred approaches, specifically the Information Systems Research framework and design thinking, for mHealth design in a developing, under-resourced context.” Few user-centred frameworks are developed in these contexts.  The description for the combined framework, and rationale for its integration using the literature, addressed the rigor required for the development of the framework (pages 7-10). The added discussion on each cycle and mode (pages 21-23) further elaborates on the success of the combined framework in a developing context. [29] emphasises the need for rigour through observations, interviews, and literature, when designing mHealth apps, which has been demonstrated in the application of the framework. |
| 2. the issue described as cited by schnall et al. with the ISR framework is an time consuming and cost intensive process. Yet, its unclear how the revised framework addresses these issues. | The revised framework involves steps such as iterative, low-fidelity prototyping with users who represented personas of end-users. These steps are contained in the design thinking techniques used, which allowed for the development of artefacts in a rapid fashion, reducing the time spent by researchers improving the mHealth app. The discussion on how the application of the framework illustrates this time-saving quality has been added on page 21:  “Through the visualisation of the interface of the app with a low-fidelity prototype, student participants could interact with the representation and identify usability concerns early in the design. They could also do this rapidly and at little to no cost to the designers. Healthcare workers could then identify how context-specific limitations would influence app usability. Time spent with healthcare workers was constrained by their busy schedules, but through the design process suggested in this paper, most of the usability limitations could be mitigated in the prior engagement with students.”  The engagement with end-users in a public healthcare setting also required less time and effort from healthcare workers, since the prototype mode advanced the progression of the design quickly, and some of its usability issues had already been addressed. In terms of cost, design thinking facilitates the creation of low-fidelity prototypes and the iteration of the design with resources and participants that are convenient to engage with. For this study, this was a cost-effective strategy, and could serve as a strategy for future mHealth projects. These insights were added to the discussion on page 21:  “Iterating with students before involving healthcare workers allowed for quick and efficient refinement of the user-interface, and further allowed for more meaningful interactions with end-users in the field. Given the above considerations, we believe that the design approach described in this paper addresses the cost and time concerns associated with the application of the ISR framework highlighted by Schnall et al.” |
| 3. We require you to either (1) present written permission from the copyright holder to publish these figures specifically under the CC BY 4.0 license, or (2) remove the figures from your submission | The letter has been obtained and uploaded with the resubmission. |

| Reviewer 1 Comments | Response to comments |
| --- | --- |
| 4. It is not clear if the approach authors have focused on is “user-centered design” or “participatory design” although they are different. It should be clearly differentiated in the introduction section and followed by that, the introduction should be re-written as it is not consistent in this manuscript. | We are aware that user-centred design involves designers and/or experts who consider the needs of the end-user throughout the design process, while participatory design includes the end-user as a member of the design or expert group. As stated in the manuscript, the focus is on user-centred design. |
| 5. The first and second paragraph should be connected. The potential of mHealth interventions in developing countries should be clear; the authors described that mHealth has the potential to increase access to health services, then mentioned it hasn’t been accompanied by the positive clinical outcomes? In the next paragraph, again to promote healthy behaviors, and then prioritizing patient and physician insights and needs? What is the success of mHealth interventions? More appropriate than what? The importance of the study should be well-stated in the introduction. | The final sentence of the first paragraph 1 has been removed in order to improve flow between paragraphs 1 and 2. The first paragraph addresses the potential of mHealth, while the second addresses mHealth design considerations. Our response to comment 1 addresses the reviewer’s comment on the importance of the study. |
| 6. The authors have claimed that “Currently, few frameworks allow for this holistic approach to mHealth app design.” To my knowledge, there are great approaches which can be used for the design of mHealth apps. Existing literature of the mHealth app design approach, usability of the mHealth interventions and evidence-based frameworks which have been used in this area should be elucidated, a problem to be addressed should be stated, and then an important need of the authors’ new framework should be proposed with the clearly stated purpose of the study. | We are aware that there are existing frameworks for mHealth app design - these have been added as references [9, 14, 15] on page 4; however, these frameworks are mostly developed and deployed in a developed context. As a result, the frameworks often lack the consideration of end-users in under-resourced and low-income contexts. This introduces multiple constraints in the design process which the proposed framework addresses. The framework is now referred to as one that is applied in an under-resourced and developing context, in the paper.  The text referenced by the reviewer has been changed to:  “Currently, few frameworks allow for this holistic approach to mHealth app design in developing and under-resourced contexts. While frameworks are available for the design of mHealth apps, few are formulated for a developing context.” |
| 7. The conceptual framework could be well-summarized in the introduction rather than the methods section, with the further rational for the combined framework, if the authors aimed to evaluate the applicability of the combined framework to their mHealth app. Or, if the purpose of the study was to report the approach, it would be good to make the manuscript containing a case study. Once again, the purpose of the study in this manuscript should be clearly stated. | Currently the conceptual framework is directly after the introduction section and does not mention the methods used in the implementation of the framework. The conceptual framework presents the theoretical underpinnings of the framework and the approach (ISR and DT) to be combined, with the implementation illustrated through use case.  Please refer to the response to comment 1 on the purpose of the study. |
| 8. The rational of the sample size and data analysis should be added. | The rationale for sample size has been moved from page 13 to page 11. This should make it clearer at the first mention of the sample size.  The rationale for the chosen data analysis has been added on page 14:  “Data analysis took the form of assertional analysis of participants’ think aloud responses. This type of analysis determines how relationships between concepts were formed during problem-solving through the verbal responses during prototyping, which was appropriate for identifying aspects of the app that needed improvement. Field observation notes were coded manually for patterns associated with the usability of the app and analysed thematically.” |
| 9. Each cycle within the proposed framework could include methods (study sample/procedures/data analysis) and results independently, which will enable the readers to avoid the confusion and enhance an understanding of using the framework for the mHealth app design. Moreover, it would be good to create a table displaying the methodologies used for each cycle/mode (e.g., think-aloud protocols/ PSSUQ). | The methods and results were reported together because of how each cycle and mode influenced the subsequent step in the design process. Often, the results of a particular step guided us to iterate or move on to the most appropriate cycle/mode of the framework. Therefore, presenting the methods and results independently might misrepresent the flow that the framework took when applied. A table showing an overview of the methods, analysis, and sampling techniques used in the application of the combined framework has been included on page 20. |
| 10. Tables of the study sample characteristics and the results of the PSSUQ should be added. | Tables are now included for the student and healthcare worker sample characteristics on pages 14 and 18, respectively. The results of the PSSUQ were presented in Figure 6. |
| 11. In the discussion section, the key findings for EACH cycle/mode could be discussed as this was to propose an approach. Moreover, the most appropriate approach in mHealth apps’ design would differ in a type/aim of mHealth apps (e.g., consumer app/ screening app), and actual end-users using the apps (e.g., patients/ health professionals), and the authors could add the in-depth discussion to set a boundary for using the combined framework for mHealth. | A discussion of each cycle and mode is now included on pages 21-23.  To address the comment on other types of mHealth apps:  “The mHealth app used in this study is intended for use as a tool in screening for latent tuberculosis infection. The app considers healthcare workers as end-users. The proposed framework would be appropriate for similar mHealth interventions. However, the expansion of the app for a diversity of applications, including consumer-based and self-monitoring mHealth apps, would require some context-specific adaptation. The emphasis of the framework on user-centred design is an advantage and broadly applicable to mHealth, given recent recognition of the benefits of end-user engagement and feedback during the mHealth lifecycle.” (p. 24, Discussion) |
| 12. Overall, this manuscript appears to be verbosely written, and the purpose of the study as well as type of study are not clearly stated. | This comment, specifically on the verbosity of the manuscript, is difficult to address as the reviewer has not indicated the sections needing improvement. The type of study is contained in the purpose of the study (please see response to comment 1). |

| Reviewer 2 Comments | Response to comments |
| --- | --- |
| 13. I feel like more quantitative evidence is needed to make it convincing that the proposed framework could be widely applicable, for example, data to show its advantages over traditionally used design methods. | We have presented a framework for a particular context. The framework may be adapted for different contexts and applications. Furthermore, the response to comment 11 addresses the broader applicability of the combined framework. |
| 14. For the case study, would the author be able to provide more detailed data on the participants’ needs that you identified in the first cycle and how they felt if their needs have been met when they tested the designed your app. | During the relevance cycle and define mode, healthcare worker needs were identified to be an efficient and comfortable app with which they could assess and evaluate the results of the TST. When the app was tested in the test mode, additional needs arose that were important to the context within which the app was used.  The following text has been added for clarity:  “The usability considerations and needs identified during initial engagement with healthcare workers were addressed by the basic functionalities of the app; however, through further engagement, additional considerations and needs arose, highlighting the importance of continuous reflexive engagement with end-users.” (p. 23)  Additional text has been added to explain the data safety needs that arose during the test mode:  “Questions of data safety also arose, relating to where the captured data would be saved and who would see it. These questions were addressed by describing who would interact with the personal medical data in the public health context (nurses, CHW, doctors, and researchers).” (p. 19) |
| 15. Could you explain why did not your include literature review in the relevance design? | The relevance cycle is where the environment of the end-user is understood. The literature review could be part of this cycle, since literature can assist in this understanding. However, the rigour cycle is more appropriate for the literature review as it is used to support what is known about the problem and environment. This puts the primary focus on engaging end-users during the relevance cycle and consolidating the findings with the literature in the rigour cycle, adding to the knowledge base. Putting the literature in the relevance cycle could shift the emphasis on end-user engagement as the primary source of identifying the problem in the specific context. Of course, since the entire framework is iterative, these cycles can occur at various stages of the design process, allowing for the literature review to intertwine with end-user understanding. |
| 16. Label of y-axes of figure 6 needs to be added. | The label has been added and the figure amended. |
