# Supplementary material for: A user-centred design framework for mHealth
Source: PLoS One. 2020 Aug 19;15(8):e0237910. doi: 10.1371/journal.pone.0237910 (PMC7444488; doi:10.1371/journal.pone.0237910)
Supplement: Attachment — Submitted filename: Response to Reviewers.docx [file pone.0237910.s002.docx]

We thank the editor and the reviewers for their valuable comments. We explain below how we have addressed them to improve the clarity of the manuscript.

| Reviewer 1 Comments | Response to comments |
| --- | --- |
| 1. In the Abstract, the Results could be improved. Some appear to be discussion/conclusion rather than results. | The Abstract, specifically the Results and Conclusion sections, has been adjusted.  The Results section reads as follows:  “The combined framework was applied through engagement with end-users, namely ten healthcare workers and ten graduate students. Through review of the literature and iterations of the app prototype, we identified various usability requirements and limitations. These included challenges related to image capture and a misunderstanding of instructions. These insights influenced the development and improvement of the app.”  The Conclusion section reads as follows:  “The combined framework allowed for engagement with end-users and for low-cost, rapid development of the app while addressing contextual challenges and needs. The integration of design thinking modes with the ISR cycles was effective in achieving the objectives of each approach. The combined framework acknowledges the importance of engaging users when implementing mHealth technologies, especially in developing and under-resourced contexts. Findings from this study support the use of this framework as a guide in the design of user-centred mHealth interventions.” |
| 2. While it says the purpose of the study is to propose a framework for the design of an mHealth app, it appears that the authors actually used the combined framework to RE-DESIGN an mHealth app for TST reading, and it was ONE case study. As the most appropriate methodological approach would differ in mHealth apps’ RE-DESIGN than in mHealth apps’ initial design process, it would be good to clarify within the entire manuscript. | The use of re-design instead of design was changed on the following pages (lines):  2 (41); 8 (172); 10 (215); 11 (228); 12 (250); 20 (398); 21 (427); 24 (495); 24 (509)  These instances relate to the specific use case in this study.  Additionally, the following text has been added for clarity:  “The framework was used in the re-design of the TST reading app but could also be used in earlier stages of mHealth app design.” (Page 2, lines 36-38)  “The framework is thus, in this case, applied to the re-design of an mHealth app. The framework is also intended for use in the early stages of mHealth app design.” (Page 4, lines 90-91)  “The integration of DT with the ISR framework results in an adapted framework which we propose for the design of mHealth apps. In this study, the framework is used in the re-design of an mHealth app, but it has broader utility in mHealth app design, including in the design of new apps.” (Page 8, lines 171-173)  “Based on the nonlinear and iterative nature of DT and the ISR framework, both approaches are suited for the design and the re-design of mHealth apps. The combined framework proposed in this study retains these features of the individual approaches. The combined framework is applied here to the re-design of an mHealth app. However, as Michl argues, design may be approached as re-design, in that “design is a sub-category in a never-ending re-design process” and “every complex product that is improved embraces a large number of clever solutions that earlier designers have contributed, and which the later designer freely adopts, makes into his own, and builds on”. The combined framework can thus be viewed as suitable both for the design and the re-design of mHealth apps.” (Page 9, lines 171-179)  “The proposed framework has been applied to the re-design of an mHealth app, in what can be considered an iteration of an existing app. While the study focuses on this single use case, the framework is also suitable for the initial design of mHealth apps since both DT and the ISR framework are iterative and non-linear, and allow for the designer’s discretion when initiating the design process, based on the needs of the project and end-user [18, 20]. The framework is especially well-suited for mHealth apps that are situated within constrained and under-resourced contexts.” (Page 24, lines 494-500) |
